# The influence of microvascular injury on T1- and T2*-relaxation times after acute myocardial infarction

**DOI:** 10.1186/1532-429X-16-S1-P211

**Published:** 2014-01-16

**Authors:** Lourens Robbers, Paul F Teunissen, Robin Nijveldt, Aernout M Beek, Maurits R Hollander, Mark B Hofman, Henk Everaars, Joost J Binnerts, Niels van Royen, Albert C van Rossum

**Affiliations:** 1 Cardiology - CMR, VU University medical center, Amsterdam, NH, Netherlands; 2 Cardiology, VU University medical center, Amsterdam, NH, Netherlands; 3 Physics and Medical Technology, VU University medical center, Amsterdam, NH, Netherlands; 4 ICIN-Netherlands Heart Institute, Utrecht, UT, Netherlands

## Background

Pre-contrast T1-mapping and Late Gadolinium Enhancement (LGE) imaging offer a detailed characterization of the infarcted myocardium and its severity after acute myocardial infarction (AMI). However, the influence of T2*-effects in infarcts with microvascular injury (MVI) on local T1-mapping values has not yet been elucidated. We evaluate the effect of T2*-decay on T1-relaxation times in the infarcted myocardium in patients after AMI.

## Methods

Forty-three patients after AMI, treated with successful primary percutaneous coronary intervention, underwent CMR at 4 (3-5) days, for cine imaging, pre-contrast T1-mapping and T2*-mapping, and LGE. MVI was defined as a contrast-devoid area in the core of the infarcted, hyperenhanced myocardium on the LGE images. T1- and T2*-mapping was performed in short-axis orientation at the level of the infarcted area. T1- and T2*- relaxation time values were determined in a region of interest (ROI) in [1] the core of the infarcted myocardium within the hyperenhanced region incorporating any area with MVI when present, [2] the border zone within the hyperenhanced myocardium but outside any areas of MVI, and [3] remote myocardium without hyperenhancement. Left ventricular volumes and ejection fraction (LVEF) were derived from the cine images and infarct size was determined on the LGE images. Normal distribution of relaxation times was achieved by log-transformation.

## Results

Twenty of the 43 patients (47%) had MVI. Infarct cores with MVI had significantly lower T1-values (MVI 1062 [974-1107]ms, vs. no MVI 1128 [1051-1185]ms, p = 0.02) and lower T2*-values (MVI 20.3 [18.2-23.3]ms vs. no MVI 30.9 [26.0-38.9]ms, p < 0.001) than infarct cores without MVI. Border zone T2*-values did not differ between patients with and without MVI; however, border zone T1-values were significantly longer in patients with MVI than in patients without MVI (MVI 1140 [1096-1174]ms, vs. no MVI 1050 [1007-1089]ms, p = 0.009). As would be expected, remote T1-values did not differ between patients with and without MVI (MVI 1000 [965-1011]ms vs. no MVI 977 [929-993]ms, p = 0.13). A significant correlation was found between T2*-values and LVEF (r = 0.60, p < 0.001), and between T2*-values and infarct size (r = -0.60, p < 0.001). For T1-values, however, these relations did not exist.

## Conclusions

Patients with reperfused AMI have shorter T1- and T2*-relaxation times in the infarct core when MVI is present. In the adjacent border zone, T1-relaxation times are longer in patients with MVI. This has important implications for the interpretation of pre-contrast T1-mapping values shortly after AMI.

## Funding

No disclosures need to be made.

